# CD163 detection in immune check-point inhibitors-related acute interstitial nephritis

**DOI:** 10.1093/ckj/sfaf009

**Published:** 2025-02-18

**Authors:** Thomas Perier, Yves Renaudineau, Juliette Pellegrini, Magali Colombat, Angie Arango Ramirez, Pierre Guy, Thibaut Jamme, Nathalie Van Acker, Clément Koundé, David Ribes, Antoine Huart, Audrey Casemayou, Julie Belliere

**Affiliations:** Department of Nephrology and Organ Transplantation, Referral Centre for Rare Kidney Diseases, French Intensive Care Renal Network, University Hospital of Toulouse, France; Laboratory of Immunology, University Hospital of Toulouse, France; Department of Nephrology and Organ Transplantation, Referral Centre for Rare Kidney Diseases, French Intensive Care Renal Network, University Hospital of Toulouse, France; Department of Pathology, Imag'IN Platform, University Hospital of Toulouse, University Cancer Institute of Toulouse, Toulouse, France; Université Toulouse, Toulouse, France; Laboratory of Immunology, University Hospital of Toulouse, France; Department of Nephrology and Organ Transplantation, Referral Centre for Rare Kidney Diseases, French Intensive Care Renal Network, University Hospital of Toulouse, France; Université Toulouse, Toulouse, France; Department of Pathology, Imag'IN Platform, University Hospital of Toulouse, University Cancer Institute of Toulouse, Toulouse, France; Department of Nephrology and Organ Transplantation, Referral Centre for Rare Kidney Diseases, French Intensive Care Renal Network, University Hospital of Toulouse, France; Department of Nephrology and Organ Transplantation, Referral Centre for Rare Kidney Diseases, French Intensive Care Renal Network, University Hospital of Toulouse, France; Department of Nephrology and Organ Transplantation, Referral Centre for Rare Kidney Diseases, French Intensive Care Renal Network, University Hospital of Toulouse, France; Department of Nephrology and Organ Transplantation, Referral Centre for Rare Kidney Diseases, French Intensive Care Renal Network, University Hospital of Toulouse, France; INSERM U1297, Institute of Metabolic and Cardiovascular Diseases, Toulouse, France; Department of Nephrology and Organ Transplantation, Referral Centre for Rare Kidney Diseases, French Intensive Care Renal Network, University Hospital of Toulouse, France; Université Toulouse, Toulouse, France; INSERM U1297, Institute of Metabolic and Cardiovascular Diseases, Toulouse, France

**Keywords:** alternatively activated macrophage, CD163, immune check-point inhibitors acute interstitial nephritis ICI-AIN, urinary soluble usCD163

## Abstract

**Background:**

Acute interstitial nephritis (AIN) is the most common renal immune-related adverse event after immune check-point inhibitors (ICI). We hypothesized that alternatively activated macrophages (CD163-M) could be involved in ICI-AIN and wished to evaluate the use of their soluble urinary form (us)CD163 as a non-invasive diagnostic marker.

**Methods:**

CD163-M infiltrates were evaluated by both immune-histochemistry and multiplex immunofluorescence and imaging. usCD163 was detected with ELLA technology and evaluated together with urinary creatinine to be expressed as a ratio to creatinuria in ng/mmol. Clinical data were collected to perform correlations with renal function assessed by estimated glomerular filtration rate (eGFR).

**Results:**

A retrospective cohort of 63 ICI-exposed patients with tubular acute kidney injury profile requiring a biopsy were selected. AIN patients (*n* = 44) were compared to acute tubular necrosis (ATN) patients (*n* = 19). CD163-M staining was detectable in all ICI-AIN patients, which was significantly higher than in ATN patients (18.4% vs 3.6% of area, *P *= .005). CD163-M staining was restricted to the interstitial compartment. CD163-M infiltrate inversely correlated with initial eGFR (*r* = −0.6, *P *= .003), and was positively correlated with delta eGFR, reflecting a renal improvement outcome (*r* = 0.48; *P *= .02). usCD163 was well detected in urines of patients, but did not allow us to distinguish ATN from AIN patients at diagnosis. No correlation was observed, neither between usCD163 and CD163-M staining nor with renal response after 3 months of glucocorticoid tapering.

**Conclusion:**

CD163-M are detected in ICI-AIN and correlate both with severity at diagnosis and better prognosis at 3 months. CD163-M may help us to distinguish AIN from ATN but, it does not allow us to assess ICI imputability. Although detected in urine, usCD163 is clearly not a surrogate biomarker for AIN diagnosis.

KEY LEARNING POINTS **What was known:**

- Data on macrophage ICI-AIN are scarce, with only four case reports describing the presence of alternatively activated macrophage (CD163-M) in the kidney.
- In a study including 35 cases of ICI-AIN, it was observed that the degree of interstitial fibrosis was the main predictor of renal outcome, but a CD163 analysis was not performed.
- Furthermore, the urinary soluble (us) form of CD163 is currently emerging as a non-invasive biomarker of renal parenchymal infiltration by CD163-M and is already used in glomerular disease.

**This study adds:**

- CD163-M staining is restricted to the interstitial compartment, detectable in 100% of ICI-AIN patients.
- CD163-M staining is inversely correlated with initial eGFR. usCD163 is clearly detected in AIN patients, but levels are not statistically significantly different from those found in ATN patients.

**Potential impact:**

- Tissular CD163-M should be considered as a target in ICI-AIN. usCD163 is not a surrogate diagnosis marker to distinguish AIN from ATN.

## INTRODUCTION

Immune check-point inhibitors (ICI) have an overwhelmingly beneficial impact on cancer treatment. At the same time, however, they confer a significant risk of developing immune-related adverse events (irAE), with acute interstitial nephritis (AIN) being the most common renal irAE. Although most studies on ICI-AIN to date have focused on adaptive immune cells and renal tertiary lymphoid structure formation [1], the key role played by innate immune cells (e.g. macrophages) deserves attention.

Abundant alternatively activated macrophage (CD163-M) infiltrations correlate with impaired renal function at biopsy and at follow-up in renal diseases [2]. Case reports describe the presence of CD163-M in kidney samples taken from ICI-AIN patients [3–5], and recent data from Farooqui *et al*., performing spatial immune cell phenotype characterization from imaging mass cytometry data, confirm the presence of macrophages in ICI-AIN [6]. CD163-M are considered to tune the inflammatory response, thereby promoting tissue remodelling or repair. However, CD163-M can also drive the fibrotic response during tissue injury. In a study including 35 cases of ICI-AIN, it was observed that the degree of interstitial fibrosis was the main predictor of renal outcome (response to treatment) [7]. Yet the authors of that study failed to establish an association between renal fibrosis/outcome and renal histological markers such as inflammation, tubulitis, the number of eosinophils and neutrophils, and the clustering or presence of CD8^+^ or CD4^+^ T cells, CD20^+^ B cells, or CD68^+^ pan-macrophages [7]. We speculate that the CD163-M subset and/or its urinary release product may reflect kidney injury in ICI-related AIN.

The urinary soluble (us) form of CD163, released by shedding, is currently emerging as a non-invasive biomarker of renal parenchymal infiltration by CD163-M. Bibliographical data on studies of large series of patients have demonstrated its interest in glomerular diseases [8–11]. Given the urgent need to develop non-invasive markers of ICI-related AIN—although some candidates, such as TNF-alpha, are promising [6]—we aimed to explore whether renal CD163-M detection on its own or coupled with urinary sCD163 detection could improve ICI-AIN management.

## MATERIALS AND METHODS

### Study design and population

In this cross-sectional and follow-up study, patients exposed to ICI, who had been referred for AKI (acute kidney injury) to the Department of Nephrology and Organ Transplantation in Toulouse University Hospital were selected. ICIs encompassed: cytotoxic T-lymphocyte antigen 4 inhibitors (ipilimumab); Programmed Cell Death Protein 1 inhibitors (pembrolizumab, nivolumab, dostarlimab); and Programmed Cell Death-Ligand 1 antibodies (atezolizumab, avelumab, and durvalumab). Patients who had an official kidney biopsy report with or without urine data available at the time of AKI were included in this study.

The study was conducted according to the guidelines of the declaration of Helsinki. Participants gave informed consent. The cohort was approved by the ethics committee in France (CPP) under the references RC31/21/0154, Molecular Prediction of Development, Progression or Complication of Kidney, Immune or Transplantation-related Diseases (Nephrogen). A further group of patients, also belonging to the Nephrogen cohort, was also selected, to provide both negative and positive controls for CD163 staining: one patient with minimal change disease and five patients with active lupus nephritis.

### Data collection

Demographic, clinical, and biological characteristics were recorded. Baseline creatinine level was defined as the last stable serum creatinine value recorded before initiating ICI therapy. AKI was diagnosed by an absolute increase in creatinine (sCr), at least 0.3 mg/dl (26.5 μmol/l) within 48 hours, or by a 50% increase in sCr from baseline within 7 days, according to the KDIGO classification [12]. We defined AIN cases and acute tubular necrosis (ATN) controls based on the diagnoses of the treating nephrologists after their review of the biopsies and based on the official biopsy interpretation from a pathologist. Urinary marker was not part of the adjudication process through which AIN was distinguished from ATN. Renal response was defined as follows: complete response was defined as return of the creatinine level to <31 μmol/l above the baseline value; partial response was defined as a return of creatinine to >31 μmol/l but less than twice the baseline value; and liberation from renal replacement therapy regardless of the creatinine value (according to Gupta *et al.* [6]).

### Macrophage detection in kidney samples

#### Immunohistochemistry

CD163 detection was automatically performed using Benchmark ULTRA (Roche, Ventana Medical Systems, Innovation Park Drive, Tucson, AZ, USA) on FFPE sections (4 μm). After deparaffinization, slides were heated using CC1 buffer (pH 8) (Roche Diagnostics) at 98°C. Slides were blocked for endogenous peroxidase activity and incubated with anti-CD163 (clone MRQ-26, Roche Diagnostics). Revelation was performed using OptiView DAB (Roche Diagnostics). Slides were counterstained with haematoxylin over 8 minutes, and Blueing reagent for 4 minutes at room temperature (Roche Diagnostic), before dehydration with ethanol and xylene. Slides were mounted using xylene-based mounting using the TissueTek coverslipper (Sakura FineTek, France). Slides were digitized with a Pannoramic 250 Flash II digital microscope (3DHISTECH, Budapest, Hungary) equipped with a Zeiss Plan-Apochromat ×20 NA 0.8 objective and a CIS VCC-FC60FR19CL 4-megapixel CMOS sensor (unit cell size 5.5 × 5.5 µm) mounted on a ×1.6 optical adapter, to achieve a scan resolution of 0.24 μm/pixel in the final image (corresponds to ×41.1 magnification at the highest optical resolution in traditional microscopy). Quantification was performed using ImageJ^®^ software (Rasband, W.S., ImageJ, US NIH, Bethesda, MD, USA) as follows: slides were digitally imaged and protein expression was quantified using plugins for quantification of percentage coverage by colour deconvolution. The threshold colour plugin was used to remove purple colour from haematoxylin-stained nuclei. A threshold without dark background was added. Images were processed so only DAB-stained portions were included in the threshold. The threshold was the same for all patients. The area of the thresholded image was measured and the final percentage of area coverage was obtained.

#### Multiplex immunofluorescence

The Discovery ULTRA (Roche, Ventana Medical Systems, Innovation Park Drive, Tucson, AZ, USA) was used to automate the staining procedure. After dewaxing, the tissue slides were heat pre-treated using a CC1 (pH 8) buffer (Roche Diagnostics) at 98°C. The slides were then stained for multiplex immunofluorescence using the RUO Discovery Universal procedure (v.0.00.0370) in a four-step protocol with sequential denaturation [CC2 buffer (pH 6), at 100°C, Roche Diagnostics] after each step. The fluorochrome sequence recommendations were respected. Tissue slides were subsequently incubated using the primary antibodies CD163 (clone MRQ-26, Roche Diagnostics), CD68 (clone KP-1, Roche Diagnostics), CD16 (clone SP175, Roche Diagnostics), and CD206-Mannose receptor (polyclonal, AB64693, Abcam Netherlands; 1/100 in Envision Flex diluent: K800621-2, Agilent Technologies, Santa Clara, CA, USA). Targets were then linked using the OmniMap anti-rabbit (Roche Diagnostics) and OmniMap anti-mouse (05269652001, Roche) HRP conjugated secondary antibodies. Visualization of the different targets was finally established using the Rhodamin6G (Roche Diagnostics), RED610 (Roche Diagnostics), Cy5 (Roche Diagnostics), and FAM (Roche Diagnostics) detection kits, respectively. The tissue slides were counterstained using Hoechst 33342 [H21492 (ThermoFisher, Waltham, MA, USA), 1/500 in Discovery Diluent (Roche)] and mounted with gelatine-mounting medium (GG1, Sigma-Aldrich, St Louis, MO, USA).

#### Tissue annotation and digital image analysis

Fluorescent-stained whole tissue slides of renal biopsies were scanned in 16 bits using the Zeiss AxioScan.Z1 (Carl Zeiss, Oberkochen, Germany) whole-slide scanner equipped with a Colibri 7 solid-state light source and appropriate filter cubes. Areas presenting artefacts such as tissue folds, degraded tissue fragments, were excluded from the analysis. Using the HighPlex FL module of the HALO imaging analysis software (Indica Labs, Albuquerque, NM, USA), we studied the macrophages in distinct regions that had been annotated by a certified pathologist. Cells stained with a cytoplasmic/membranous staining intensity exceeding the settings threshold were counted as positive for the full range of staining intensity. Results were represented as the percentage of the cells that were positive for the defined phenotypes.

### Kidney histopathology scores

Histology analysis was performed blind by two independent pathologists. Interstitial fibrosis and tubular atrophy (IF/TA) were expressed as percentage of the total area (fibrosis in %). The tubular injury score was determined based on the percentage of tubules showing luminal casts, cell detachment, or dilation and assigned according to the following scale to assess tubule-interstitial fibrosis: 0 = 0 to 5%; 1 = 6 to 25%; 2 = 26 to 50%; 3 = 51 to 75%; and 4 > 75%. Arteriosclerosis classification was the following: cv0 = no chronic vascular changes; cv1 = vascular narrowing of up to 25% of the luminal area by fibrointimal thickening; cv2 = vascular narrowing of 26% to 50% of the luminal area by fibrointimal thickening; and cv3 = vascular narrowing of >50% of the luminal area by fibrointimal thickening. Arterial hyalinosis was evaluated as follow: aah1 = replacement of degenerated smooth muscle cells by hyaline deposits in only one arteriole, without circumferential involvement; aah2 = replacement of degenerated smooth muscle cells by hyaline deposits in more than one arteriole, without circumferential involvement; and aah3 = replacement of degenerated smooth muscle cells by hyaline deposits with circumferential involvement, independent of the number of arterioles involved.

### Urinary sCD163 detection

The spot urinary sCD163 (ELLA^®^, Bio-techne, Minneapolis, MN, USA) was evaluated together with urinary creatinine (Cobas 500*^®^*) to express usCD163 as a ratio to creatinuria in ng/mmol, as previously described [13].

### Statistical analysis

Statistical analyses were performed with GraphPad software. Mann–Whitney and Kruskal–Wallis were used. Correlations were calculated using Spearman coefficient. A *P* value of <.05 was considered as significant. Summary statistics were presented as mean (SD) for continuous normally distributed variables, median (interquartile range) for continuous variables with skewed distributions, and as *n* (%) for categorical variables. Comparisons of measures between ATN and AKI groups were evaluated using the equal variance *t*-test for normally distributed variables, the Wilcoxon rank sum test for non-normally distributed variables, the chi-squared test for categorical variables where the expected cell counts were >5, and the Fisher exact test for categorical variables where the expected cell counts were <5.

## RESULTS

### Clinical characteristics of selected patients

**Figure 1:**
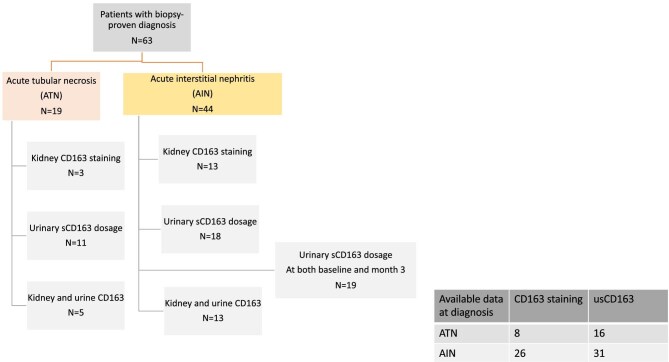
Flowchart of the study.

We retrospectively selected a cohort of 63 patients receiving ICI and experiencing AKI with a biopsy-proven diagnosis of ATN (*n* = 19) or AIN (*n* = 44) (flow chart in Fig. 1). Clinical characteristics of patients are detailed in Table 1. Most patients were treated for lung adenocarcinoma. Frequencies of hypertension, diabetes, and chronic kidney disease (defined as eGFR < 60 ml/min/1.73 m^2^) before AKI were similar in both groups, as well as ICI type, mainly anti-Programmed Cell Death Protein 1. As anticipated, proton-pump inhibitors frequency was significantly higher in AIN than ATN group (43.2% vs 10.5%, *P *= .018), whereas exposure to platinum-based chemotherapy was higher in ATN than AIN (89.5% vs 61.4%, *P *= .036). Extra-renal irAEs were reported in 26% of ATN and 74% of AIN patients without reaching statistical significance (*P *= .77). Admission for AKI occurred after a median number of five (minimum 1 to maximum 32) ICI injections. Initial eGFR was lower in AIN than in ATN patients (27 vs 37 ml/min/1.73 m^2^, *P *= .03). Leucocyturia was more frequent in AIN than in ATN (61.4% vs 21.5% of patients, *P *= .005).

**Table 1:** Patients’ characteristics.

	ATN *n* = 19	AIN *n* = 44	Total *n* = 63	*P* value
Baseline parameters				
Male gender, *n* (%)	12 (63.2)	24 (54.5)	36 (57.1)	.59
Age at time of AKI (y), mean (SD)	63 (±8)	66 (±11)	65 (±10)	.2
Malignancy treated with ICI, *n* (%)				.21
Lung adenocarcinoma	16 (84.2)	23 (52.3)	39 (61.9)	
Melanoma	1 (5.3)	9 (20.5)	10 (15.8)	
Renal cell carcinoma	0 (0)	2 (4.5)	2 (3.2)	
Ovarian carcinoma	0 (0)	1 (2.2)	1 (1.6)	
Others	2 (10.5)	9 (20.5)	11 (17.5)	
Comorbidities, *n* (%)				
Hypertension	6 (31.6)	15 (34.1)	21 (33.3)	.9
Diabetes mellitus	1 (5.3)	5 (11.4)	6 (9.5)	.66
Basal creatinine (μmol/l), median (min–max)	74.5 (58–105)	80 (48–220)	80 (48–220)	.31
eGFR <60 ml/min/m^2^ before treatment, *n* (%)	0	8 (18)	8 (12.7)	.09
ICP type, *n* (%)				.30
Anti-PD1	16 (84.2)	37 (84.1)	53 (84.1)	
Anti-PDL1	2 (10.5)	1 (2.3)	3 (4.8)	
Anti-PD1 combined with anti-CTLA4	1 (5.3)	6 (13.6)	7 (11.1)	
Comedications, *n* (%)				
PPI	2 (10.5)	19 (43.2)	21 (33.3)	.018
NSAID	1 (5.3)	1 (2.3)	2 (3.2)	.52
Antibiotics	1 (5.3)	10 (22.7)	11 (17.5)	.15
Iodine contrast products	0 (0)	5 (11.3)	5 (7.9)	.31
Platinum-based chemotherapy	17 (89.5)	27 (61.4)	44 (69.8)	.036
Nb of injections before AKI, median (min–max)	4 (1–9)	6 (1–32)	5 (1–32)	.08
At admission for AKI				
Creatinine (μmol/l), mean (SD)	233 (±234)	247 (±141)	243 (±173)	.77
eGFR (ml/min/1.73 m^2^), mean (SD)	37 (±16)	27 (±14)	30 (±16)	.02
Haematuria, *n* (%)	5 (26.3)	24 (54.5)	29 (46)	.05
Leucocyturia, *n* (%)	4 (21.5)	27 (61.4)	31 (49.2)	.005
Hypereosinophilia, *n* (%)	3 (15.8)	5 (11.4)	8 (12.7)	.69
extra-renal IRAe, *n* (%)	5 (26.3)	14 (73.7)	19 (30.2)	.77
Biopsy characteristics				
Number of glomeruli, median (min–max)	16 (8–31)	15 (5–35)	16 (5–35)	.15
Percentage of sclerotic glomeruli, median (min–max)	4 (0–25)	7 (0–54)	7 (0–54)	.60
Percentage of IFTA area, median (min–max)	5 (1–10)	15 (5–80)	10 (1–80)	.0001
Presence of granuloma (%)	0 (0)	5 (11.4)	5 (7.9)	.31
ATN, *n*	19	33	0.16	
No (%)	1 (5.3)	3 (9.1)		
>25%	7 (36.8)	12 (36.4)		
25%–50%	0 (0)	5 (15.2)		
50%–75%	5 (26.3)	11 (33.3)		
>75%	6 (31.6)	2 (6.1)		
Vascular fibrous intimal thickening, *n*	19	41	0.82	
cv0 (%)	10 (52.6)	18 (43.9)		
cv1 (%)	3 (15.8)	11 (26.8)		
cv2 (%)	5 (26.3)	12 (29.3)		
cv3 (%)	1 (5.3)	0 (0)		
Arteriolar hyalinosis, *n*	19	40		.66
aah0 (%)	12 (63.2)	28 (70)		
aah1 (%)	5 (26.3)	6 (15)		
aah2 (%)	0 (0)	5 (12.5)		
aah3 (%)	2 (10.5)	1 (2.5)		
AIN evolution				
Glucocorticoids exposure (weeks), median (IQR)	N/A	10 (1–26)		
Renal response at 3 months, *n* (%)	N/A	39		
Complete	N/A	18 (46.1)		
Partial	N/A	19 (48.7)		
No response	N/A	2 (5.1)		

PD1, Programmed Cell Death Protein 1PDL1, Programmed Death-Ligand 1; CTLA4, Cytotoxic T-Lymphocyte Associated Protein 4; PPI, proton-pump inhibitor; NSAID, non-steroidal anti-inflammatory drugs; IFTA, interstitial fibrosis and tubular atrophy. N/A, not applicable.

### Histopathological characteristics of kidney biopsies

Detailed parameters are available in Table 1 as well as Supplementary Table S1. Despite being statistically higher in AIN than in ATN patients (15% vs 5%, *P *= .0001), the median percentage of fibrosis was low, suggesting an acute process. Granuloma were exclusively found in AIN biopsies. Then, ATN, vascular fibrous intimal thickening and arteriolar hyalinosis scores were similar between ATN and AIN patients.

### CD163-M macrophage detection in ICI-AIN kidney tissues

**Figure 2:**
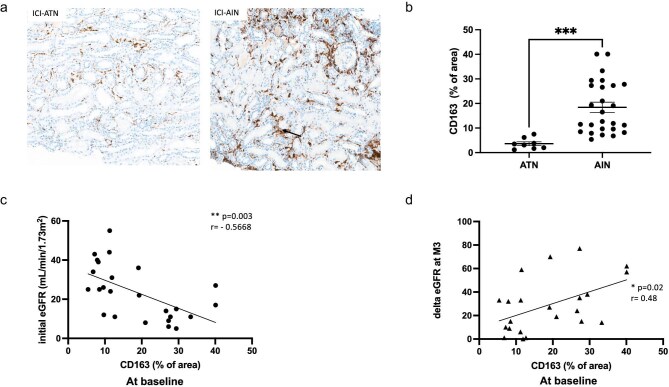
CD163-(M) macrophages in ICI-AIN kidney biopsies. CD163 immunohistochemistry detection in kidney biopsies. (**a**) A representative example is depicted for both ATN and AIN patients, in the left and right panels, respectively. (**b**) CD163 staining, expressed in percentage of area in each group (*n* = 8 for ATN and *n* = 26 for ICI-AIN) *P *= .0005. (**c**) CD163 infiltration was associated with severity of ICI-AIN at diagnosis (a high CD163-M staining was found to be associated with a low eGFR, *n* = 25, **P = .003). (**d**) CD163-M infiltration at ICI-AIN diagnosis was associated with a favourable delta eGFR at 3 months (delta = eGFR at 3 months; eGFR at AIN diagnosis), *n* = 22, *P = .02.

CD163-M immunochemistry staining was performed on 34 patients (*n* = 8 with ATN and 26 with AIN). To assess CD163-M staining, biopsies from one patient with minimal change disease (MCD, negative control) and five patients with active lupus nephritis (LN, positive control), were selected. CD163-M staining was minimal in the kidney from the MCD patient compared to the active LN patients, who presented strong CD163-M staining both in glomeruli and interstitial compartments (Supplementary Fig. S1). In both ATN and ICI-AIN patients, CD163-M staining was restricted to the interstitial compartment (Fig. 2a). In AIN patients, CD163-M were much more abundant than in ATN patients (18.4 vs 3.6, *P *= .005) (Fig. 2b). CD163-M were detectable in 100% of ICI-AIN patients. In most cases, the CD163-M infiltrate was more important than lymphocytes (Supplementary Table S1). To detect macrophage subtypes, we performed a multiplex analysis on two patients using four markers: CD16 as monocyte marker, CD163 and CD206 as M2 markers, and CD68 as a pan-macrophages marker. As shown in Supplementary Fig. S2, CD163 was well detected (25% of area) and slightly higher than CD206.

### Correlation between CD163-M infiltration and renal outcome in AIN patients

CD163-M staining was inversely correlated with initial eGFR (*r *=* −*0.57; *P *= .003, Fig. 2c), suggesting that an elevated recruitment of CD163-M could impair renal function. All patients then received steroid treatment tapered over a median of 12 weeks, and renal responses were the following: complete response (46%); partial response (49%); and no response (5%) (Table 1). To assess whether initial CD163-M staining could predict renal outcome, delta eGFR (= eGFR at 3 months—eGFR at AIN diagnosis) was calculated. Initial CD163-M staining was positively correlated with delta eGFR, reflecting renal improvement outcome (*r* = 0.48; *P *= .02) (Fig. 2d). No correlation was observed between CD163-M infiltration and histological parameters (Supplementary Fig. S3), neither with severity of fibrosis (expressed as percentage of area), nor with sclerosis of glomeruli (expressed as percentage of observed glomeruli). CD163-M infiltration repartition between AIN patients was similar, regardless of their tubular injury or arteriosclerosis or hyalinosis scores.

### Urinary usCD163 is detected in ICI-AIN patients at diagnosis but similarly to ICI-ATN patients

**Figure 3:**
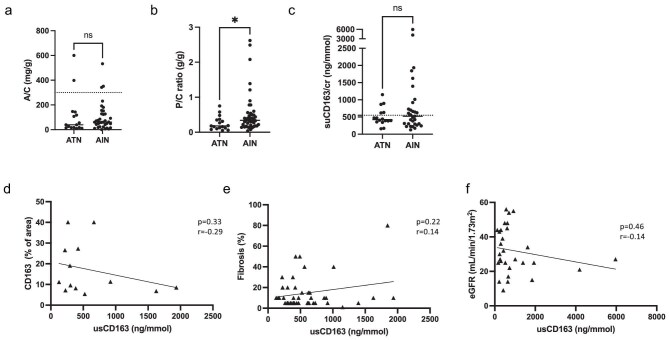
usCD163 detection in patients with ICI-AIN. (**a**) Albuminuria/urinary creatinine (A/C) ratio, expressed in mg/g. Threshold at 300 mg/g is indicated with horizontal line. (**b**) Proteinuria/urinary creatinine (P/C) ratio, expressed in mg/g. (**c**) usCD163 correlation with CD163 staining. (**d**, **e**, **f**) usCD163 correlation with degree or fibrosis and severity of AKI (initial eGFR).

Because usCD163 interpretation may be disturbed by abundant albuminuria, or glomerular proteinuria, both albuminuria/creatinine (A/C) and proteinuria/creatinine (P/C) ratios were carefully monitored in our cohort. A/C ratio was similar and very low in both groups [median 39 (9–600) mg/g in ATN, vs. 61 (0–533) mg/g in AIN, *P *= .45] (Fig. 3a), whereas median P/C ratio was significantly higher in AIN than in ATN patients [0.34 (0.04–2.62) vs 0.18 (0.05–0.75) mg/g, *P *= .02] (Fig. 3b), suggesting a low-molecular weight proteinuria rate. Altogether, A/C and P/C ratios were in the expected range for correctly evaluating usCD163. To assess whether usCD163 could be used as an AIN biomarker, usCD163 quantification was performed in 31 patients with biopsy-proven ICI-AIN and 16 patients with ATN (Fig. 3c). usCD163 levels in AIN patients were similar to those obtained in ATN patients. Median values were 424 (min 157–max 1151) ng/mmol in ATN patients, compared to 523 (min 125–max 5965) ng/mmol in AIN patients (*P *= .57). When employing the usual clinical threshold used for glomerular diseases, fixed at 525 ng/mmol in our settings with the ELLA second-generation detection kit, 48% (15 out of 31) of patients would have been considered to be positive in the AIN group compared to 31% (5 out of 16) of patients in the ATN group. Contrary to reports in glomerular diseases [7, 10, 11], usCD163 did not correlate with CD163 staining, either in AIN patients (*n* = 13, Fig. 3d) or in ATN patients (*n* = 5, data not shown). Neither the degree of fibrosis nor the severity of AKI (initial eGFR) were associated with usCD163 (Fig. 3e, f). It is worth noting that no difference was identified in usCD163 levels according to ICI type (data not shown).

### usCD163 kinetic variations

**Figure 4:**
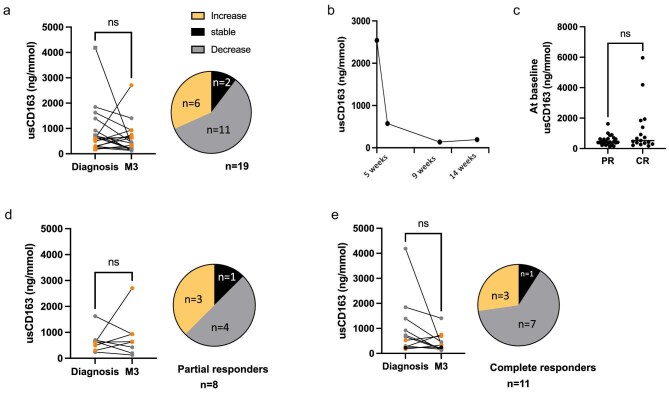
UsCD163 kinetic across AIN evolution in patients with available samples and renal response after 3 months (M3) of glucocorticoids (*n* = 19). (**a**) Individual values are plotted at diagnosis and M3. The proportion of patients with stable, increase, or decrease values is represented in a pie chart. (**b**) An illustrative example is depicted for a patient with longitudinal usCD163 monitoring and complete renal response. (**c**) UsCD163 values are depicted at diagnosis, in patients with PR compared to CR. (**d**) Patients in PR only (*n* = 8). Individual values are plotted at diagnosis and M3. Proportion of patients with stable, increase, or decrease values is represented in pie chart. (**e**) Patients in CR only (*n* = 11). Individual values are plotted at diagnosis and M3. The proportion of patients with stable, increase, or decrease values is represented in a pie chart.

We then specifically focused on patients with available samples and renal response after 3 months of glucocorticoids (*n* = 19). While usCD163 remained stable in two patients (11%) and significantly increased in six patients (32%), the most common kinetic was a decrease, observed most patients (58%) (Fig. 4a). An individual kinetic is illustrated in Fig. 4b. Because the patient with the individual kinetic obtained a rapid and complete renal response, we investigated whether usCD163 could be used to predict partial (PR) or complete (CR) renal response. First, we looked at usCD163 values at diagnosis in patients with PR and CR at M3 (Fig. 4c), finding that there was no difference in initial usCD163 between two groups. UsCD163 was not predictive of renal response. We then assessed usCD163 evolution in each group and observed that in both PR and CR patients there was no specific behaviour of the marker (Fig. 4d). Thus, usCD163 significantly varies with the course of AIN treatment, but it is unlikely that renal response was related to such kinetics.

## DISCUSSION

Here, we argue in support of the detection of CD163-M in the interstitial compartment in ICI-AIN patients, thus expanding on previous case reports [3]. We confirm that in ICI-AIN patients, renal CD163-M staining is strongly and significantly increased compared to ATN. CD163 may help in distinguishing AIN from ATN, but it does not allow us to assess ICI imputability, as has been observed in ICI-dependent [14] or independent contexts [15]. CD163 infiltration correlates with renal severity at diagnosis, which supports the hypothesis that kidney failure might by partially controlled by the recruitment of CD163 positive cells (encompassing dendritic cells and monocytes, which co-express this receptor). The reported positive correlation between initial CD163 and eGFR recovery at 3 months is further consistent with the fact that glucocorticoids repress CD163-M pro-inflammatory properties [16].

As ICI-AIN patients exhibited strong CD163-M infiltrates, we tested the performance of the usCD163 biomarker in ICI-AIN diagnosis and follow-up. Because CD163 molecular weight (MW) is 125 kDa, thus far superior to albumin MW, glomerular leak of the protein through the damaged glomerular filtration barrier is unlikely in our cohort, which exhibits low levels of albuminuria. Our data suggest that:

- usCD163 was detected in urine samples from both ATN and AIN patients, with values superior to the threshold used for glomerular diseases in 15 out of 31 AIN patients, raising the need of further investigations to fix a relevant threshold in the context of tubular injury.
- usCD163 did not correlate with CD163 staining, suggesting a spatial restriction to the interstitium or a relative retention by glomeruli. usCD163 has been demonstrated to reflect renal CD163-M in glomerular diseases, as it is enzymatically cleaved by the inflammation-responsive protease ADAM17 [9]. CD163 cleavage by ADAM17 may vary in the presence of interstitial fibrosis and tubular atrophy [17], which deserves further study. usCD163 kinetic obeys a tri-modal repartition with the course of AIN (stable, increase, decrease) that seems to be independent of renal response. It has been previously suggested that CD163-M encompass distinct macrophage subtypes, playing opposite roles in kidney repair. Using a single surface marker to capture renal prognosis is challenging, and a forthcoming single-cell dataset on ICI-AIN will provide greater detail on macrophage subtypes [18].

Clearly, usCD163 is not the surrogate non-invasive diagnosis or prognosis marker for ICI-related AIN. Interpreting CD163 also requires consideration of the GC effect on CD163 *per se* [19, 20]. Further studies are needed to evaluate the potential of other candidates, such as CXCL9, RBP/creatinine ratio [21, 22], and TNFa [6]. The sensitivity, specificity, and positive likelihood ratio of the combination of the most promising candidates (CXCL-9, TNFa, and RBP/creatinine) still needs to be examined.

## CONCLUSION

CD163 macrophages are detected in kidney biopsies in ICI-AIN and correlate both with severity at diagnosis and better prognosis at 3 months. usCD163 failed to distinguish ATN from AIN patients, and varies with AIN treatment independently of the renal response. Further studies are required to assess its added value in combination with other biomarkers, e.g. while defining macrophage infiltrate with in-depth technologies.

## Supplementary Material

sfaf009_Supplemental_Files

## Data Availability

The data underlying this article are available in the article and in its online supplementary material. The data underlying this article will be shared on reasonable request to the corresponding author.
