# Supplementary material for: CD163 detection in immune check-point inhibitors-related acute interstitial nephritis
Source: Clin Kidney J. 2025 Feb 18;18(3):sfaf009. doi: 10.1093/ckj/sfaf009 (PMC11883220; doi:10.1093/ckj/sfaf009)
Supplement: sfaf009_Supplemental_Files [file sfaf009_supplemental_files.zip › Supplemental Table 1.docx]

| Patient | CD3 | CD4 | CD8 | CD4/CD8 ratio | CD20 | CD68 | CD163 |
| --- | --- | --- | --- | --- | --- | --- | --- |
| 1 | + | + | + | 2/3 | + | + | ++ |
| 2 | ++ | ++ | + | 2/3 | + | + | ++ |
| 3 | ++ | ++ | + | 2/3 | + | + | ++ |
| 4 | ++ | + | + | 1/2 | + | + | +++ |
| 5 | ++ | ++ | ++ | 2/3 | + | + | +++ |
| 6 | ++ | ++ | ++ | 2/3 | + | + | ++ |
| 7 | ++ | ++ | + | 2/3 | + | ++ | ++ |
| 8 | ++ | ++ | + | 2/3 | + | ++ | +++ |
| 9 | ++ | ++ | + | 2/3 | + | + | ++ |
| 10 | ++ | ++ | + | 2/3 | ++ | ++ | +++ |
| 11 | ++ | ++ | ++ | 2/3 | + | + | +++ |
| 12 | ++ | ++ | + | 2/3 | + | + | +++ |
| 13 | ++ | ++ | + | 3/4 | + | + | +++ |
| 14 | ++ | ++ | ++ | 1/2 | + | + | ++ |
| 15 | ++ | ++ | + | 2/3 | + | + | +++ |
| 16 | ++ | ++ | + | 2/3 | + | + | +++ |
| 17 | ++ | ++ | + | 3/4 | + | + | +++ |

**Supplemental Table 1. Semi-quantification of immune populations in AIN kidney biopsies.** CD3 is used as surface marker for lymphocytes, CD4 for T helper, CD8 for cytotoxic T cells, CD20 for B-cells, CD68 for pan-macrophages, and CD163 for macrophages. Proportion of CD4 compared to CD8 is expressed as “CD4/CD8 ratio”. Staining was semi-quantified by two independent pathologists.
