# Supplementary figures and images for: CD163 detection in immune check-point inhibitors-related acute interstitial nephritis
Source: Clin Kidney J. 2025 Feb 18;18(3):sfaf009. doi: 10.1093/ckj/sfaf009 (PMC11883220; doi:10.1093/ckj/sfaf009)

A.

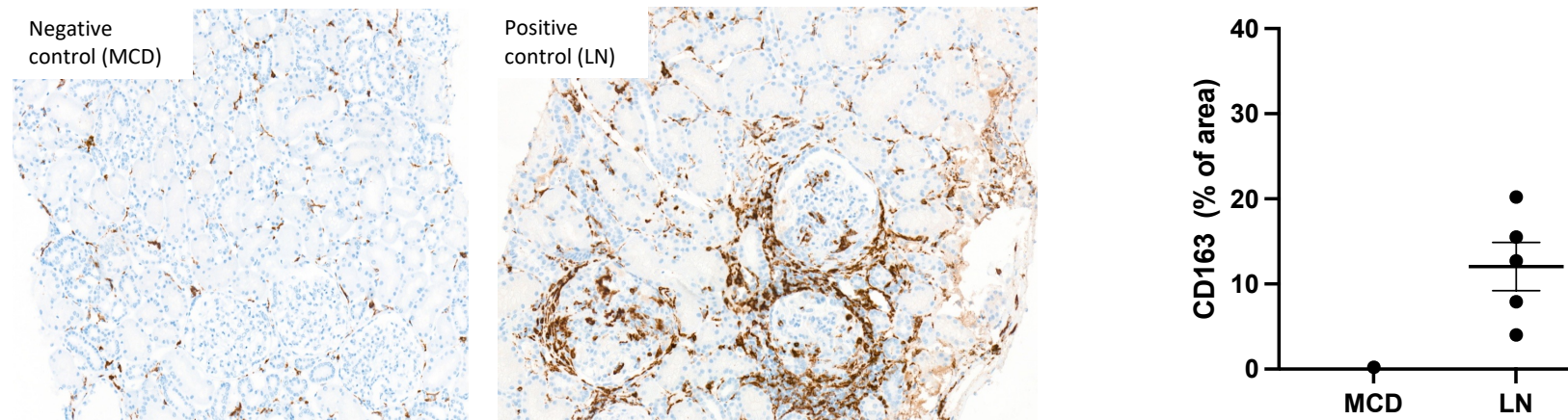

Suppl Figure 1

A.

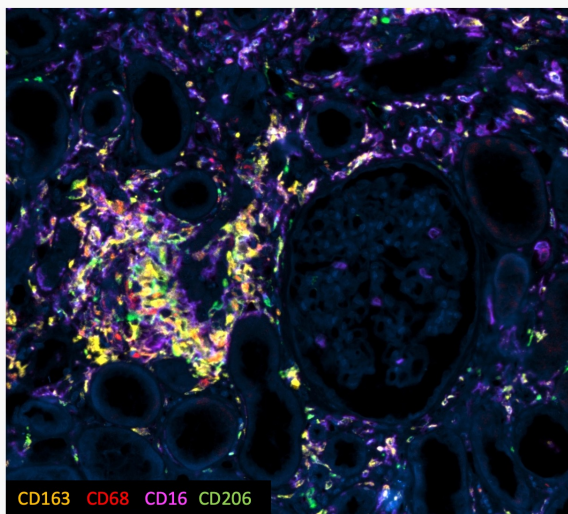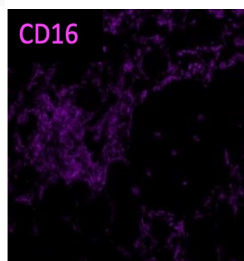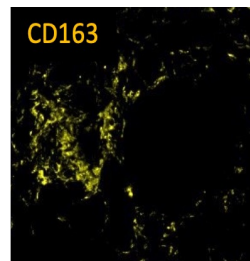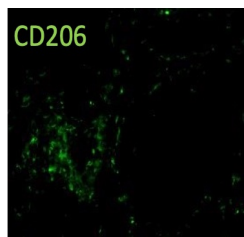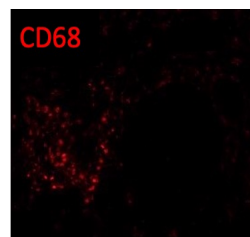

B.

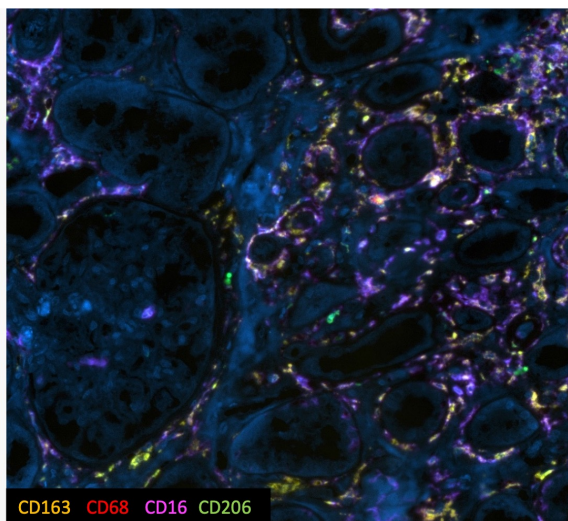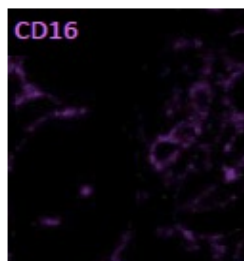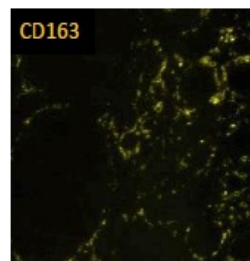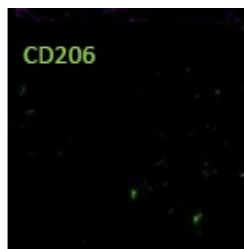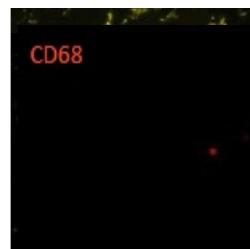

C.

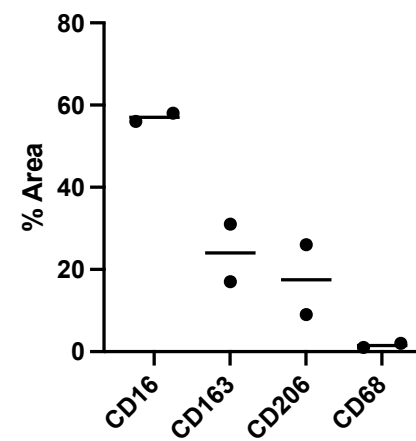

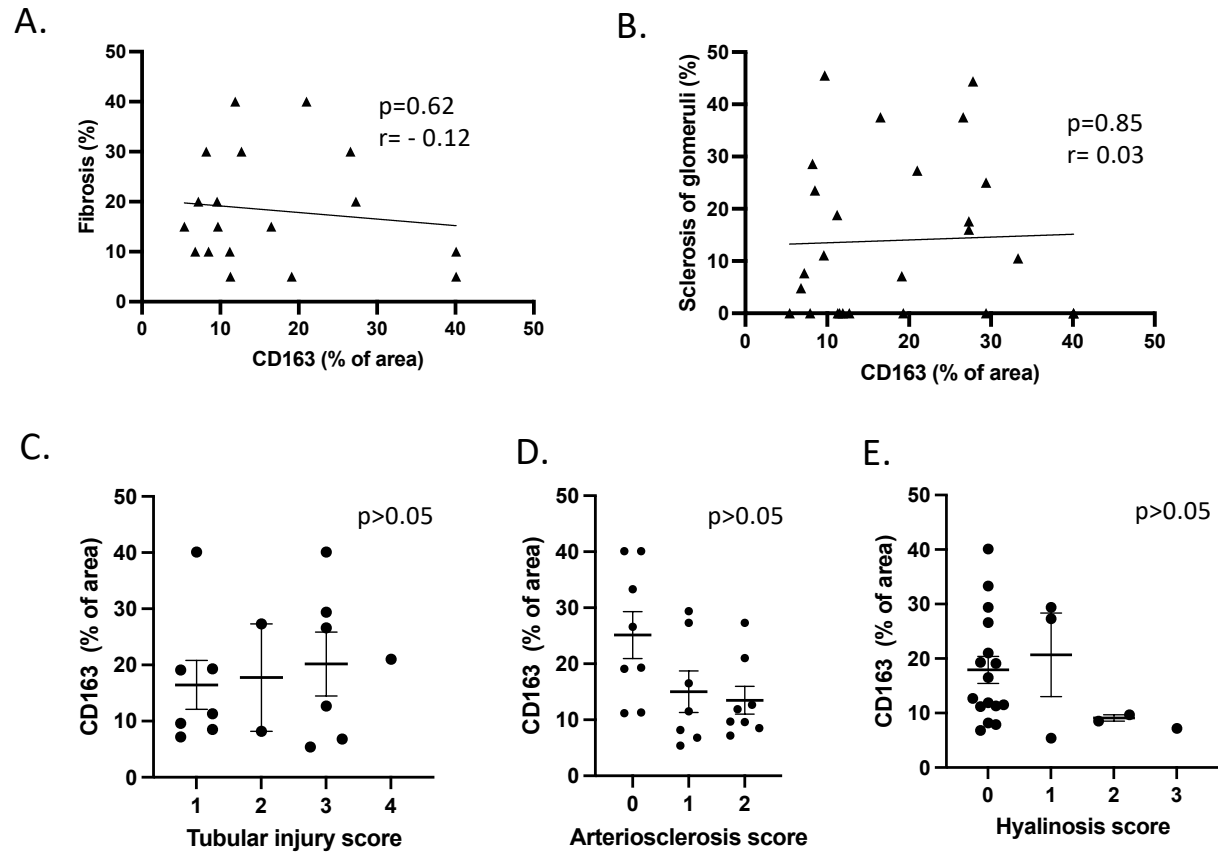

Suppl Figure 3.

Supplement: sfaf009_Supplemental_Files [file sfaf009_supplemental_files.zip › SupplementaryFIG.pdf]
